# Some culture is hiding in plain sight in research on child development

**DOI:** 10.1111/bjdp.70053

**Published:** 2026-05-31

**Authors:** Roman Stengelin

**Affiliations:** ^1^ Department of Comparative Cultural Psychology Max‐Planck‐Institute for Evolutionary Anthropology Leipzig Germany; ^2^ Department of Psychology and Social Work University of Namibia Windhoek Namibia

**Keywords:** contextualization, cross‐cultural psychology, generalizability, global north, minority world, WEIRD

## Abstract

Child development is cultural in nature, yet a divide persists between a (cross‐)cultural developmental science niche alongside a seemingly a‐cultural mainstream. In particular, childhood research relying on convenience sampling in often Western, post‐industrial (i.e., WEIRD) societies rarely ventures into issues of culture and context, promoting excessive overgeneralization and false assumptions of what is considered ‘typical’ in child development. This article has two aims: First, it illustrates the cultural make‐up of the communities traditionally overrepresented yet often unmarked in developmental science, while highlighting important variations within this cluster. Second, it demonstrates how this cultural make‐up, in turn, is key to the methods employed, the findings produced and the theories postulated in developmental science. Regardless of where it is conducted, developmental science is a cultural endeavour and thus needs to grapple with its cultural foundations.

**Statement of Contribution**

**What is already known on this subject?**

- Research and theory in human development remain overly focused on children from urban, post‐industrial communities in North America and Western Europe.
- Research in these communities rarely contextualizes its participants, instead engaging in broad generalization while neglecting its cultural foundations.

**What does this article add?**

- This article illustrates the common cultural make‐up of societies traditionally overrepresented in developmental research, while highlighting diversity within them.
- This cultural make‐up typically goes unnoticed in the communication and interpretation of child development research, yet it is foundational to the questions asked and findings obtained.

Fish are born expecting water, and humans are born expecting culture.

–Mike Tomasello

There are these two young fish swimming along and they happen to meet an older fish swimming the other way, who nods at them and says ‘Morning, boys. How's the water?’ And the two young fish swim on for a bit, and then eventually one of them looks over at the other and goes ‘What the hell is water?’

–David Foster Wallace, *This is Water*.

Childhood is key for the construction of the human mind. It is cultural in nature, shaped by and enabling the breadth of human behaviours and experiences found worldwide (Bjorklund, 2018; Rogoff, 2003). Foundational theories of human development have long acknowledged the fundamental importance of culture on developmental processes and outcomes (Bronfenbrenner, 1979; Vygotsky, 1978).

Despite this, empirical data and theory in developmental science exhibit a significant bias towards particular childhoods, often exclusively relying on convenience sampling in Western, Educated, Industrialized, Rich and Democratic (i.e., WEIRD) communities (Henrich et al., 2010). Over 90% of publications in child development research originate from North America, Europe, Australia, or Aotearoa New Zealand (Nielsen et al., 2017). This skewed sampling goes hand in hand with the widespread practice of treating these participants as a culture‐free default that requires little contextualization: here, culture remains *unmarked* (Medin et al., 2010; see also Causadias et al., 2018). As a result, research conducted in traditionally overrepresented communities often assumes, explicitly or implicitly, that its findings and conclusions generalize broadly (Cheon et al., 2020; DeJesus et al., 2024 ). In contrast, research in traditionally underrepresented communities—whether they are countries in the Global South or minoritized communities within the Global North—is *marked* explicitly and thus localized to the specific community under study (Causadias et al., 2018; Medin et al., 2010; Stengelin et al., in press).

## CHILD DEVELOPMENT IS NEVER FREE OF CULTURE

A foundational assumption in cognitive and psychological science is that human minds are structurally uniform and universal (Kroupin et al., 2025; Levinson, 2012). Developmental scientists may be particularly prone to this tenet, assuming that research with children, infants in particular, safeguards against or minimizes eventual cultural influence.

However, over the past decades, research has repeatedly shown that even seemingly fundamental aspects of child development, including milestones, sequences and timetables, are subject to significant cultural variation. For example, extensive research has documented that around age three, many children not only imitate others' instrumental or functional behaviours but will also copy actions irrelevant to achieving instrumental outcomes. This ‘over‐imitation’ (Horner & Whiten, 2005; Lyons et al., 2007) is widespread among children growing up in post‐industrial societies where social learning often takes place within formalized pedagogy (Hoehl et al., 2019). In contrast, research in subsistence‐based communities with minimal emphasis on formal schooling finds children considerably less prone to over‐imitate (Berl & Hewlett, 2015; Stengelin et al., 2019; see also Lancy, 2010).

As another example, false belief understanding is widely considered a litmus test for Theory of Mind development. Children across diverse post‐industrial societies typically begin to understand and verbalize that others' behaviours will be misguided by their false beliefs around age four, a developmental shift replicated across numerous studies and societies (Wellman et al., 2001). However, these societies often share an emphasis on mental states in verbal discourse, encouraging children to pay attention to subjective mental states and equipping children with high levels of psychological autonomy (Kärtner, 2015). In societies lacking such cultural training, children often master false belief tasks at much later ages with considerable interindividual variation in task performance (Dixson et al., 2018; Mayer & Träuble, 2013).

Another example concerns children's self‐regulation for delaying gratification as shown in the marshmallow task (Mischel et al., 1989). In post‐industrial societies, preschoolers typically struggle to delay gratification for future benefits, and their tendency to master such scenarios predicts adjustment much later in life (Shoda et al., 1990). For Cameroonian Nso children, who grow up in social systems characterized by hierarchical relatedness rather than psychological autonomy, delaying gratification appears much less challenging (Lamm et al., 2018; see also Koomen et al., 2020).

Together, these observations suggest that certain developmental milestones may appear robust across different, even diverse, societies within and beyond the WEIRD cluster. However, these societies share key cultural features and institutions, such as access to formal schooling or an emphasis on children's psychological autonomy. In fact, their historic overrepresentation in child development research may, in part, stem from these shared cultural characteristics. As a result, convergence in research findings from these societies can create universality assumptions that may collapse once assessed across more varied cultural contexts.

Notably, cultural variation in child behaviour and experience is already evident in infancy (e.g., Geangu et al., 2016; Green et al., 2016; Streeter, 1976; Wormann et al., 2014) and for seemingly ‘fundamental’ domains such as perception, attention, spatial cognition and motor development (Bremner et al., 2016; Gislén et al., 2003; Haun & Rapold, 2009; Karasik et al., 2010; Tu et al., 2025). This is critical because children's performance in developmental research, particularly in infancy and early childhood studies, often relies on these domains. For example, differences in social attention and motor skills determine how infants and toddlers respond to experimental stimuli in common research paradigms, such as eye‐tracking and reaching preference tasks.

## CHILD DEVELOPMENT RESEARCH REQUIRES DIVERSIFICATION *AND* CONTEXTUALIZATION

Without a doubt, the diversification of developmental science is thus essential for a globally relevant understanding of human child development and behaviour (e.g., Moriguchi, 2022; Nielsen et al., 2017; Rad et al., 2018; Singh, Basnight‐Brown, Cheon, et al., 2025). Still, publications from and with historically underrepresented communities remain rare (Singh et al., 2023; Singh, Basnight‐Brown, Cheon, et al., 2025; Thalmayer et al., 2021). Recently, scholars have provided practical guidance on obtaining and reporting socioeconomic data (Singh, Barokova, Baumgartner, et al., 2024; Singh, Barokova, Bazhydai, et al., 2024). However, psychological scientists remain hesitant to incorporate such efforts into their workflows, often prioritizing the quantification of focal variables over collecting sociodemographic variables.

Participant diversification and the inclusion of sociodemographic measures are essential avenues for child development research. Yet, a common barrier is that researchers often do not know how to begin without disrupting their research workflows. What is needed is raised awareness of culture as it is hiding in plain sight in much of developmental science—from the participants we study to the methods we employ—and practical guidance on how to mark the cultural peculiarities of such research.

The goal of this article is not to introduce new arguments or data to debates on participant diversity in developmental science—such points have been convincingly made elsewhere (Broesch et al., 2023; Causadias et al., 2018; Draper et al., 2023; Nielsen et al., 2017; Singh, Basnight‐Brown, Cheon, et al., 2025). Just like some fish unaware of the waters surrounding them, culture is easily overlooked by those immersed in it. Yet, child development is cultural in nature (Rogoff, 2003; White et al., 2018) and is key even for those who think it is not for them. Thus, this article aims to provide an entry point and resource to those starting to engage with the cultural foundations of their work.

## BARRIERS TO CULTURAL PERSPECTIVES IN CHILD DEVELOPMENT RESEARCH

This requires an understanding of the practical constraints faced by researchers who cannot travel the globe to test child participants but instead rely on convenience sampling in their local communities. Developmental scientists rightfully seek to optimize resource efficiency by relying on established infrastructures such as local research laboratories, personnel and participant databases. Under such conditions, the motivation for concentrated diversification efforts remains limited (Syed, 2025), especially if such shifts would disrupt existing workflows. Additionally, a lack of formal training in (cross‐)cultural psychology (Ridley, 1985; Stengelin et al., 2024) further dissuades engagement with issues of culture and context. Such issues likely pose a subjective threat to research agendas built around convenience sampling in traditionally overrepresented communities, as many ‘choose not to care about culture because of the fear to venture into the unknown or the desire to maintain status quo’ (Wang, 2016, p. 584). Under these circumstances, not only is the diversification of developmental science limited, but even the explicit contextualization of its participants is relegated to a niche with dedicated journals, issues, reviewers and editors. Note also that valuable research questions remain unanswered and extant findings require replication in the often post‐industrial communities from which they originate.

At the same time, the epistemological limitations and ethical issues inherent in a lack of diversity in developmental science necessitate authors to acknowledge the cultural environments hosting and shaping their research. Homogeneous convenience sampling, where participants are selected to share similar sociodemographic characteristics, can yield precise estimates for these target demographics (Jager et al., 2017). However, this requires that research findings are contextualized and generalized to these narrow communities. Scholars recruiting participants exclusively in Western, post‐industrial communities and specific sociodemographic communities therein need to recognize that their research questions, methodologies, findings and implications are culturally influenced.

## UNMARKED CULTURES IN CHILD DEVELOPMENT RESEARCH

Next, I review common sociodemographic makeups of children and their families typically participating in developmental science and their models of caregiving and child development. These models are noteworthy in that they are both historically recent and globally rare, adapted for the post‐industrial livelihoods hosting most of child development research. Yet, they are typically *unmarked* in scientific communication (Medin et al., 2010; Stengelin et al., in press). A summary of cultural features common in these societies with clear relevance to child development is provided in Figure 1 below.

**FIGURE 1:**
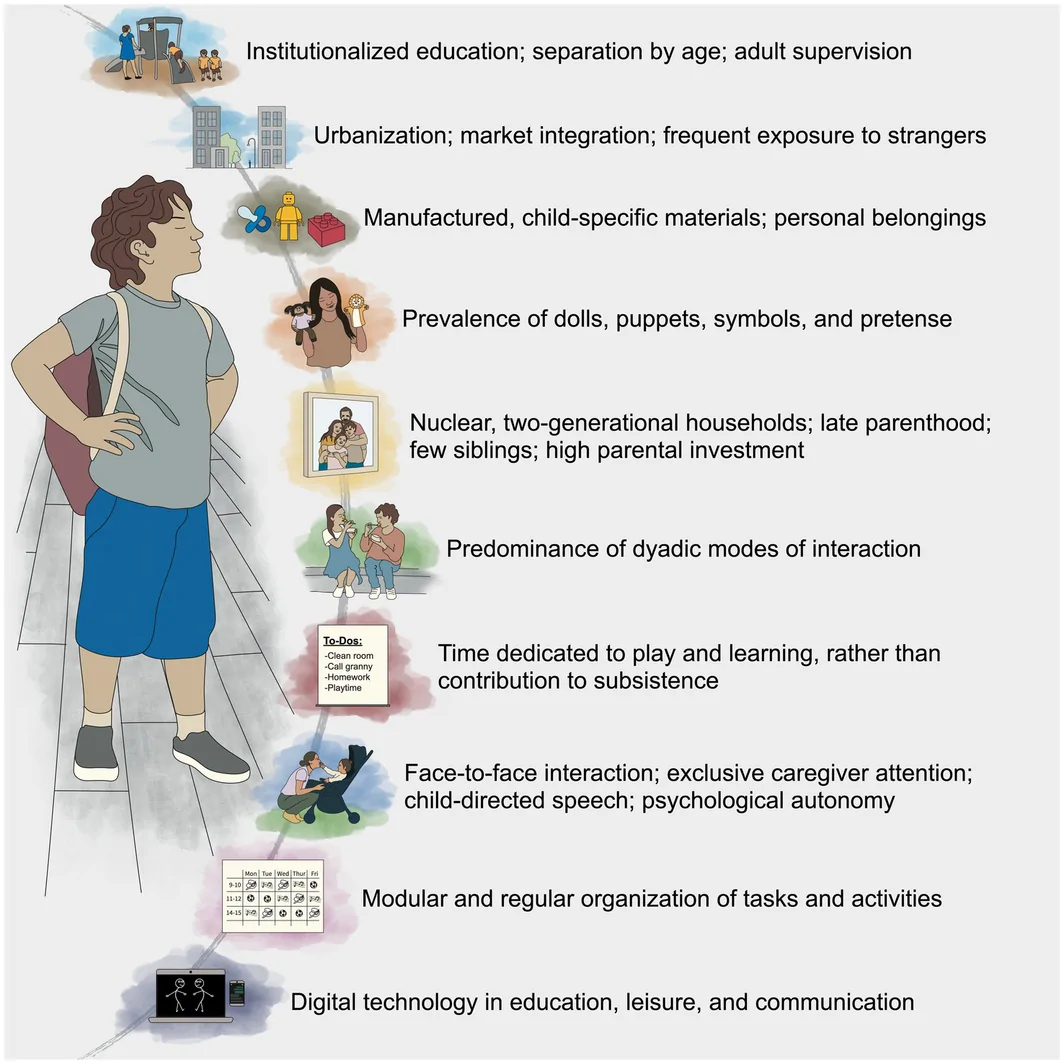
Culture Hiding in Plain Sight in Child Development Research. Common socio‐cultural variables in communities typically recruited in child development research; these variables are often unmarked in scientific communication, yet they are key to the methods, results and conclusions in such research.

Developmental science typically recruits caregivers and children from predominantly white, urban, upper‐middle class communities within Europe (i.e., the United Kingdom, Germany, the Netherlands), North America (i.e., the United States, Canada), or Oceania (i.e., Australia, Aotearoa New Zealand). Despite being among the most extensively researched communities in developmental science, scholars working within these regions are the least likely to research culture and contextualize their participants (Stengelin et al., in press). Thus, these nations, or the majority societies therein, serve as key references below. Note, however, that many of the issues discussed extend to other societies commonly sampled for convenience, including some (i.e., ‘non‐WEIRD’) post‐industrial East Asian societies (Krys et al., 2025).

## UNMARKED DEMOGRAPHICS IN CHILD DEVELOPMENT RESEARCH

Child participants in developmental science often come from rather homogenous communities when compared to other global communities, but also within the regions from which they are recruited. Families with exposure to *secondary and tertiary education* typically participate (Clancy & Davis, 2019; Doebel & Frank, 2022; Fernald, 2010) as these demographics are accessible to scholars working at research institutions. Children from immigrant or minoritized communities are often underrepresented or even excluded from research participation, particularly when recruitment criteria rely on factors such as mother tongue or monolingualism (Dawson‐Hahn et al., 2024). Moreover, individuals from communities historically overrepresented in such research are likely to show interest and consent to research participation (Yu et al., 2020). Such communities are easier to approach in research conducted at educational institutions (i.e., science museums, zoos) and are eager to invest personal efforts (i.e., travelling) to participate in lab‐based research.

Children typically grow up in *small, nuclear, two‐generational households* comprising one to three children and one or two adult caregivers (United Nations Population Division, 2022a). Caregivers, often children's biological parents, start families in their mid‐to‐late twenties or thirties, much later than the global average (United Nations Population Division, 2022b). Children in these communities typically have *one or two siblings*, reflecting country demographics with low birth rates and small proportions of children within overall population structures. Parents hold primary responsibility for childrearing. Child fostering within extended family networks is relatively rare. Grandparents and other relatives may contribute to childrearing, but daily interactions and responsibilities often remain within the nuclear family. Beyond this nucleus, kinship is not the key determinant of social life (Henrich, 2020). Instead, social exchange involves friends, colleagues and also transient, short‐term encounters with strangers (e.g., on playgrounds).

From toddlerhood, *institutional daycare* becomes normative (UNICEF, 2019). This professionalized approach to early childhood education emphasizes learning as a key developmental task assigned to young children while their caregivers participate in a service‐based economy. Children and their parents go separate ways during much of the day, leaving the morning and afternoon hours for family matters and routines. At schools and childcare institutions, children follow *set curricula* to maximize learning progress under adult tuition. Learning content is iteratively and modularly organized following set curricula. Children engage in relatively homogeneous peer groups regarding age and skill level.

Daily activities beyond daycare and schooling are also often organized externally. Afternoon activities, such as sports or music lessons, aim to cultivate children's talents and skills. The choice of activity often involves negotiation between children and caregivers, balancing children's interests with caregivers' educational goals. Consequently, these activities are often instructor‐led and viewed as educational investments (Ochs & Kremer‐Sadlik, 2015).

Children participating in research often stem from *mid‐to‐high socioeconomic backgrounds*, residing in *urban areas* with universities and colleges. These communities are characterized by social and economic security, with healthcare systems ensuring regular medical and psychological screening (United Nations, 2021). Households are integrated into market and service‐based economies, necessitating parental participation in the labour force, which further incentivizes institutional childcare to supervise and educate children. *Indo‐European languages*, particularly English, dominate (Blasi et al., 2022; Majid & Levinson, 2010).

From an early age, children are exposed to *digital technology*, such as smartphones, tablets and TV screens (UNICEF, 2017). This media serve educational, communicative and entertainment purposes tailored to children's interests and demands. This includes cartoons, often featuring anthropomorphized animals or objects agents as characters and repetitive plot structures (e.g., resolving tension, benevolent protagonists outcompeting malevolent antagonists), often serving as positive reinforcement. Besides digital media, children are surrounded by *objects made for them*, such as toys, books and furniture (Ochs & Kremer‐Sadlik, 2015).

## UNMARKED CHILDREARING IN CHILD DEVELOPMENT RESEARCH

Caregivers share some unifying themes in child‐rearing, such as individual achievement, independence, positive self‐esteem, (psychological) autonomy, self‐regulation and creativity (Alwin, 2001; Harkness et al., 2007; Keller & Kärtner, 2013; Markus & Kitayama, 1991; Nomaguchi & Milkie, 2019). For example, caregivers often value individualism as they encourage children to become *independent, self‐sufficient agents* (Hofstede, 1980; Triandis, 1989). Tamis‐LeMonda et al. (2008; see also Ryan & Deci, 2000) identify four prevalent values in this milieu: emphasis on personal choice, intrinsic motivation and persistence, self‐esteem and self‐maximization. Following these values, caregivers aim to acknowledge and promote children's personal skills and talents, rather than their social responsibilities in family or society.

*Autonomy* is a key socialization goal (Kagitcibasi, 2005; Keller, 2012; Keller & Otto, 2011) but remains largely restricted to the psychological domain (i.e., *psychological autonomy*; Keller, 2012; Keller & Otto, 2011): children are encouraged to navigate social interactions based on their needs and desires rather than social obligations (Ochs & Kremer‐Sadlik, 2015; Zelizer, 1994). Their action autonomy, reflected in independent and self‐initiated contributions to chores and household activities, is less valued. Childhood is considered a *phase of learning and play*, rather than contribution and obligation. Involvement in chores is rare and voluntary (Keller, 2007, 2012) and considered a prosocial act (Giner Torréns & Kärtner, 2017), whereas obedience and responsibility is de‐emphasized (Nomaguchi & Milkie, 2019). Caregivers and children engage on almost‐equal footing: children have a say in many aspects of daily life, such as food choice or family activity. This focus mirrors the ideals of a *democratized parent‐child relationship* (Ochs & Kremer‐Sadlik, 2015).

The focus on psychological autonomy does not imply that another fundamental social need—relatedness—holds little value (Kagitcibasi, 2005; Keller, 2012). Children are encouraged to navigate social interactions based on personal choice and intrinsic motivation, often leading to transient and flexible engagements with peers and adults. Exchange of positive emotions is common, both for peer‐ and adult‐child interactions: ‘the conception of relatedness is in the service of psychological autonomy’ (Keller, 2016b, p. 2), and social interaction is idealized as a source of pleasure and personal fulfillment.

Society values *authoritative parenting* in which caregivers are highly responsive to children's needs while setting reasonable limits. Authoritative parenting promotes secure and exclusive attachment, so children form close bonds with one (often their mother) or a few individual caregivers (Keller, 2016a). This emphasizes the role of dyadic relationships (Dayton et al., 2022; Rogoff, 2003) and affective intimacy with exclusive individuals, yielding positive consequences on various developmental outcomes (Keller, 2016a; Otto & Keller, 2014). Physical punishment, such as spanking, is forbidden by law and negatively affects development (Smetana, 2017). Instead, caregivers are encouraged to direct child behaviour through positive reinforcement and by establishing a mutual understanding of impartial norms and adequate, responsible behaviour.

Caregivers invest considerable cost and effort into childrearing. For example, they guide children's learning and attention to relevant and age‐appropriate content, sharing visual attention through ostensive cues while altering language and behaviours (Little et al., 2016; Shneidman & Woodward, 2016). They simplify activities and demands to ease children's contribution and experience of success. Children's achievements are highlighted and rewarded with praise and material incentives. Caregivers commonly engage in activities and games designed for children, tailoring behaviours and language to reduce imbalances in competence. They aim to accommodate and promote children's perspectives, putting them centre stage. Such *child‐centrism* is also reflected in high monetary (Kornrich & Furstenberg, 2013) and time investments (Pew Research Center, 2013) for early childhood care and education.

Caregivers' primary style of engaging with children is *distal*, providing verbal instruction within *face‐to‐face settings* and *object stimulation* (Keller & Kärtner, 2013). Caregivers, mostly mothers, address children verbally, and child‐directed speech increases with age (Bergelson et al., 2019). Beyond child‐directed speech, caregivers elicit children's smiling and frequently mirror children's affect (Keller & Otto, 2009), promoting happiness as an ideal affective state while sensitizing children to their own intentionality and experience (Kärtner, 2015). This is further supported by parental mind‐mindedness, whereby caregivers verbally reflect upon children's mental states, such as their beliefs, desires and emotions (Meins et al., 2002). Further training in mental state language and thought is provided though storybooks (Farkas et al., 2020; Okumura et al., 2024). Together, such practices foster children's *sensitivity to mental states*, promoting self‐awareness and emphasizing the subjectivity of human experience.

Children are encouraged to pursue their interests and become self‐sufficient. At school, they learn abstract skills, including literacy and algebra, as these skills are considered crucial in later life. The ideal of ‘the entrepreneurial child’ (Ochs & Kremer‐Sadlik, 2015), innovative and competent at solving novel problems, guides parenting. Merit‐based fairness norms determine resource allocation in social exchange (Schäfer et al., 2015, 2023), preparing children for their economic and cultural realities. Children are raised to develop *analytical thinking*, navigating the social and objective world with an emphasis on focal objects and their attributes, abstract rules and formal logic rather than broader contexts, relationships and harmony (Nisbett et al., 2001).

## CULTURAL VARIATION WITHIN TRADITIONALLY OVERREPRESENTED SOCIETIES

Despite such commonly shared patterns, these communities exhibit much diversity within. Developmental science typically recruits participants from societies on the individualist side of the global spectrum, and increasingly from individualist communities within societies historically considered more collectivistic (Santos et al., 2017). Yet, much variation also prevails within Western, post‐industrial societies: while the United States, the United Kingdom and Australia rank highest in global individualism metrics, mainland European countries including Germany or the Netherlands, but also Canada, score markedly lower on this dimension (Hofstede, 2011). The United States is also characterized by substantially higher ratings in religiosity (Joshanloo & Gebauer, 2020); economic inequality (World Bank Open Data, 2022); and ethnic, linguistic and religious fractionalization (Alesina et al., 2003; see Wormley et al., 2022, for further cultural metrics) compared to most European countries, but also Australia or Canada. Social constructions relating to race and ethnicity vary further between the United States and other countries, urging for nuanced contextual grounding when reporting on sampling approaches and participant demographics (Jugert et al., 2022; Roberts et al., 2020; Singh et al., 2023).

Additionally, factors such as the availability of social security systems, paid parental leave policies and the age at which children enter institutional daycare vary significantly both across and within countries in this cluster. Given these differences, it is not surprising that cross‐cultural research within the Western hemisphere has found considerable variation in the behaviours and preferences of caregivers and children (e.g., Harkness et al., 2007; Harkness & Super, 2006; Suizzo, 2007; Suizzo & Bornstein, 2006).

There are evident disparities in how research from specific communities *within* countries is typically interpreted and applied (Causadias et al., 2018; Roberts et al., 2020). Research conducted within minoritized communities in the United States, for example, has established a strong tradition of contextualizing findings within the broader societal landscape (e.g., Alcalá et al., 2018; Coll et al., 1996; Corriveau et al., 2017; Zapata Roblyer et al., 2015). These studies demonstrate that attention to cultural context and the study of cultural variation in child development do not require scholars to conduct research abroad. Moreover, in‐depth analyses of cultural variation are also available for communities traditionally overrepresented in child development research (Kärtner, 2015; Ochs & Izquierdo, 2009; Ochs & Kremer‐Sadlik, 2013, 2015; Otto & Keller, 2015).

Taken together, developmental science has traditionally relied upon testing children in communities clustering on multiple cultural metrics critical for child development. This includes, but is not limited to, the factors encapsulated in the WEIRD backronym (Henrich et al., 2010). Where such clustering occurs, participants often represent the extremes of global cultural metrics with clear relevance to children's lived experiences. At the same time, these communities also exhibit significant diversity in children's and caregivers' everyday experience. Both patterns underscore the importance of carefully contextualizing the communities in which child development research takes place—*especially* when working with communities traditionally overrepresented, yet often unmarked in scientific publications.

## THE CULTURAL FOUNDATIONS OF RESEARCH METHODS IN CHILD DEVELOPMENT

Given the historic overreliance on participants from these communities, it is not surprising that research on child development is both tied to and tailored to them. This includes the questions developmental scientists tend to ask (Atari et al., 2025) and the findings they discover, as well as the paradigms and approaches routinely employed.

To establish causality in child development, developmental scientists often strive for individual testing in standardized settings. For example, experimental control often requires the involvement of adult experimenters. Children often engage with strangers one‐on‐one, mimicking the adult‐structured settings that our typical participants experience frequently. Such scenarios often focus on dyadic settings, employing turn‐taking conversations with children responding to pedagogical questioning, closely resembling children's experience in schools and at home (Keller et al., 2019; Stengelin et al., 2026). Cultural training in symbolism, depictions of real‐world objects, pretence and hypothetical thinking prepares children for research protocols utilizing such elements to evoke responding, such as simplified figurines and depictions of agents and objects (Kominsky et al., 2022).

Hierarchies between children and adults are easily de‐emphasized to ensure that children comfortably express themselves within the research context, thereby revealing their cognitive skills and preferences. Children regularly establish rapport and trust with unfamiliar adults, promoting social engagement after brief warm‐up phases in playful, reciprocal modes (Barragan & Dweck, 2014). While researchers encourage children to act ‘naturally’ in study settings, their attention is often directed to the tasks at hand, restricting their behaviours and attention spatially and thematically. Adults provide critical instructions and supervision while engaging with children on an almost‐equal footing to render study participation rewarding and participatory. In one approach, for example, puppets are effectively employed to lower perceived hierarchies between children and adults akin to peer interactions (Rakoczy, 2022; Stengelin, Ball, et al., 2023; Stengelin, Haun, & Kanngiesser, 2023). Children's psychological autonomy allows them to navigate social encounters based on their preferences without much considering prevailing social bonds. If children are tested within well‐established relationships, such as with classmates, friends, or their caregivers, these encounters are typically marked by positive affect and cooperation.

Developmental scientists frequently employ technological devices to structure and standardize research protocols. Children raised in technology‐saturated societies are comfortable with video instruction and conferencing (Fong et al., 2021; Sommer et al., 2023) and are used to being video‐recorded. They are frequently exposed to complex, causally opaque devices and technology, handling such objects confidently with or without adult tuition. Being exposed to such material culture in research implies that free exploration, handling and innovation are permitted and encouraged, likely leading to positive reinforcement (e.g., fun experience, material rewards).

## CONTEXTUALIZING RESEARCH

Taken together, the demographics and parenting practices inherent to the communities typically studied in child development research critically shape the questions, paradigms and findings of the discipline. Children carry their cultural experience and assumptions to the culturally matched tasks when participating in developmental science. Thus, providing information about the cultural context hosting the research is as essential as other methodological information to comprehend, reproduce, replicate and generalize child development research (Syed, 2023).

However, the field lacks unified standards and guidance on how to grapple with culture (Singh, Barokova, Bazhydai, et al., 2024). Two steps can be easily undertaken to move forward in this. First, researchers should systematically contextualize their participants in the context of their research. Such efforts are increasingly mandated by academic publishers: the *Society for Research in Child Development*, for example, demands authors to provide ‘the theoretically relevant characteristics of the particular sample studied (e.g., race/ethnicity, socioeconomic status, native language, gender identity, religion)’ as well as ‘the place(s) from which that sample was drawn, including country, region, city, neighborhood, school, etc. and all other context variables that are relevant to the focus of the publication’ (Society for Research in Child Development, 2023). *Developmental Science* and *Infancy* require information on participant demographics, taking a ‘flexible approach as to the specific indicators used’ (Developmental Science Author Guidelines, 2025; see also Oakes, 2025). Such information may include sample demographics directly obtained from participants (Singh, Barokova, Baumgartner, et al., 2024; Singh, Barokova, Bazhydai, et al., 2024), but ethnography and publicly available data may also provide relevant information.

Finally, sub‐disciplines within child development, such as research on minoritized childhoods or neighbourhood effects on child development, offer valuable lessons in situating participants within broader cultural communities (Fuller & García Coll, 2010; White et al., 2021; Witherspoon et al., 2023). This includes individual‐level assessments of participants' cultural orientation (Knight et al., 2018) as well as group‐level proxies for children's cultural experience (Amir & McAuliffe, 2020; Causadias, 2013; Knight et al., 2018). Combining assessments of culture on both levels, researchers can gain a more nuanced understanding of how cultural variation in child experience and behaviour unfolds ‘one child at a time’ (Bohn et al., 2024; see also Knight et al., 2023).

Beyond mandates for standardized contextual information, relating research paradigms and questions to children's cultural livelihoods can critically inform readers and scientific communities about the research. Similar to information on research design, materials, or analyses, such information can be provided in the methods sections of scientific communication (e.g., under *participants*). Further, researchers need to communicate and elaborate on the generalizability of their research (Kroupin et al., 2025; Yarkoni, 2022). If sampling is constrained to specific, relatively homogenous communities—which is typically the case in psychological research (Jager et al., 2017)—the cultural characteristics and boundaries of these communities need to be clearly defined and made explicit. Generalizability assumptions depend on sampling *and* research outcomes and are thus ideally communicated prominently in the discussion sections.

## CULTURE IN DESCRIBING RESEARCH

### Demographics

First, researchers must provide structured information on participant demographics. Singh, Barokova, Baumgartner, et al. (2024) propose a framework encompassing various demographic categories known to influence developmental outcomes. This includes biological information, gestational status, health status, community of descent, caregiving environment and socioeconomic status (Singh, Barokova, Baumgartner, et al., 2024). Researchers are encouraged and increasingly mandated to include assessments of these categories in their workflows.

They may also use publicly available resources on the country‐ or community level. For example, information on average household sizes, formalized education and family income can be gleaned from official reports or surveys at the community or country level or international surveys. Such macro‐level data is naturally limited in explaining and predicting individual‐level data (Amir & McAuliffe, 2020; Pollet et al., 2014), yet it can help situate participants and communities within the target community.

Demographics, such as social status and information about children's home environments, are key to understanding and predicting social, cognitive and physical development both across and within societies. Yet, such information is only rarely provided in research outputs (Singh, Barokova, Baumgartner, et al., 2024; Singh & Rajendra, 2024). Scholars can enrich their research by providing such information when describing the participants under investigation.

### Research design and question

In the study of child development, research design relies on children's cultural competencies. Researchers can enhance transparency and communicative precision by reflecting on the cultural assumptions and prerequisites of their procedures. For example, researchers employing video instructions might describe children's experience with such devices and forms of communication. Similarly, scientists testing children's causal understanding with unfamiliar, causally opaque devices could discuss children's exposure to adult‐introduced technology and the adequacy of exploration in such settings. Prior research in similar communities using similar approaches strengthens arguments for cultural appropriateness and transferability, but any detail, even if anecdotal, helps to contextualize participants and scientific approaches.

Beyond the role of culture in study design, researchers can situate their research questions (Atari et al., 2025). A unified approach to this is challenging due to the vast diversity of research topics. However, researchers may follow a rule of thumb: identify critical independent and dependent variables, often those stated in abstracts. Next, consider (cross‐cultural or mono‐cultural) research within and outside WEIRD communities investigating these variables to understand their potential cultural impact. Relevant research with adult participants may also provide valuable information. For example, research employing minimal group manipulations may consider discussing the role of natural groups in their participants, such as ethnic fractionalization or social stratification, or the role of self‐enhancement in culture (Falk et al., 2014). Studies on social learning may document participants' exposure to schooling and formalized pedagogy (Lancy, 2016; Lew‐Levy et al., 2020), and research on Theory of Mind development may consider the socialization of mental state language (Taumoepeau et al., 2019) or how mental states are recruited for moral judgement or applied in daily exchange (Barrett et al., 2016).

By design, research approaches in developmental science embody cultural assumptions and knowledge about the participants under study. Children bring their cultural concepts, experience and thinking to the laboratory, shaping their behaviours and responses in developmental research. Communicating such information is key to interpreting and generalize findings to other cultural communities and to enabling direct or conceptual replications as a cornerstone of an open and responsible research on child development.

## CULTURE IN DISCUSSING RESEARCH

All developmental research seeks to generalize to some extent, yet explicit discussions of these assumptions are rare. To date, researchers are incentivized to remain strategically ambiguous by applying findings broadly (e.g., to a generic ‘children’) without stating or justifying such assumptions. This convention hinders replication and theoretical advancement. By incorporating dedicated ‘Constraints on Generality’ sections (Simons et al., 2017), researchers can clarify the scope of their research based on established contextual information. This creates a dedicated space to, for example, discuss how task suitability might vary across cultures, informing future conceptual replication. Also, such sections can discuss whether similar findings would be expected in cultural communities varying across relevant cultural metrics.

Kroupin et al. (2025) suggest generalizing findings within a cultural evolution framework, specifying the ‘Environment of Articulation’ in which the effects under investigation are assumed to apply and recur (see also Henrich, 2008). Whether or not assumptions are discussed in dedicated sections or as part of the general discussion needs to be addressed by editorial boards and journals, but any explicit communication of generalizability increases transparency and is preferable over strategic ambiguity.

## CULTURE IN CHILD DEVELOPMENT RESEARCH: RESEARCH AND TEACHING INSTITUTIONS

Situating child development research culturally requires efforts from the scientists who conduct and publish the research. However, meaningful change requires parallel efforts from universities and research institutions. In many educational systems and institutions, psychological training rarely and unsystematically engages with culture and diversity as core components of the curriculum (Ridley, 1985; Stengelin et al., 2024). Adopting perspectives from related disciplines, such as anthropology or sociology, is not necessarily part of student training in psychology or related fields.

Without systematic training, hiring and funding, research institutions are likely to cultivate developmental science as a field that seeks to bypass its cultural foundations. Thus, anchoring culture and diversity more firmly in psychology requires a shift in the incentives and priorities set out by scientific institutions as well as the funding bodies and academic journals serving as gatekeepers to the research that is conducted and taught (Syed, 2025).

## CONCLUSION

A global science of child development necessitates diversification of participants and researchers. However, a significant portion of research continues to originate from Western, post‐industrial societies. Such research, including its questions, methods and conclusions, is tied to the cultural communities hosting it. It is time we actively recognize the culture that is hiding in plain sight in developmental science.

## AUTHOR CONTRIBUTIONS

**Roman Stengelin:** Conceptualization; writing – original draft.

## CONFLICT OF INTEREST STATEMENT

The author has no conflicts of interest to declare.

## Data Availability

Data sharing not applicable to this article as no datasets were generated or analysed during the current study.
